# Enhancing Left Ventricular Segmentation in Echocardiograms Through GAN-Based Synthetic Data Augmentation and MultiResUNet Architecture

**DOI:** 10.3390/diagnostics15060663

**Published:** 2025-03-09

**Authors:** Vikas Kumar, Nitin Mohan Sharma, Prasant K. Mahapatra, Neeti Dogra, Lalit Maurya, Fahad Ahmad, Neelam Dahiya, Prashant Panda

**Affiliations:** 1 CSIR-Central Scientific Instruments Organisation (CSIR-CSIO), Chandigarh 160030, India; vikas.csio18j@acsir.res.in (V.K.); nitin.csio17a@acsir.res.in (N.M.S.); 2 Academy of Scientific and Innovative Research (AcSIR), Ghaziabad 201002, India; 3 Anaesthesia and Intensive Care, Postgraduate Institute of Medical Education and Research, Chandigarh 160012, India; neeti_dogra@rediffmail.com (N.D.); drneelamdahiya@gmail.com (N.D.); prashantpanda85@gmail.com (P.P.); 4 School of Computing, University of Portsmouth, Portsmouth PO1 3HE, UK; fahad.ahmad@port.ac.uk; 5 Portsmouth Artificial Intelligence and Data Science Centre (PAIDS), University of Portsmouth, Portsmouth PO1 3HE, UK

**Keywords:** echocardiograms, data augmentation, Generative Adversarial Networks, MultiResUnet, segmentation

## Abstract

**Background**: Accurate segmentation of the left ventricle in echocardiograms is crucial for the diagnosis and monitoring of cardiovascular diseases. However, this process is hindered by the limited availability of high-quality annotated datasets and the inherent complexities of echocardiogram images. Traditional methods often struggle to generalize across varying image qualities and conditions, necessitating a more robust solution. **Objectives**: This study aims to enhance left ventricular segmentation in echocardiograms by developing a framework that integrates Generative Adversarial Networks (GANs) for synthetic data augmentation with a MultiResUNet architecture, providing a more accurate and reliable segmentation method. **Methods**: We propose a GAN-based framework that generates synthetic echocardiogram images and their corresponding segmentation masks, augmenting the available training data. The synthetic data, along with real echocardiograms from the EchoNet-Dynamic dataset, were used to train the MultiResUNet architecture. MultiResUNet incorporates multi-resolution blocks, residual connections, and attention mechanisms to effectively capture fine details at multiple scales. Additional enhancements include atrous spatial pyramid pooling (ASPP) and scaled exponential linear units (SELUs) to further improve segmentation accuracy. **Results**: The proposed approach significantly outperforms existing methods, achieving a Dice Similarity Coefficient of 95.68% and an Intersection over Union (IoU) of 91.62%. This represents improvements of 2.58% in Dice and 4.84% in IoU over previous segmentation techniques, demonstrating the effectiveness of GAN-based augmentation in overcoming data scarcity and improving segmentation performance. **Conclusions**: The integration of GAN-generated synthetic data and the MultiResUNet architecture provides a robust and accurate solution for left ventricular segmentation in echocardiograms. This approach has the potential to enhance clinical decision-making in cardiovascular medicine by improving the accuracy of automated diagnostic tools, even in the presence of limited and complex training data.

## 1. Introduction

Cardiovascular disease (CVD) is a significant concern globally and the leading cause of death worldwide [1]. It is closely linked with multiple factors, including the left ventricular ejection fraction (LVEF). LVEF measures the changes between the left ventricular end-diastolic volume (EDV) and end-systolic volume (ESV), with a lower LVEF being indicative of a worse outlook [2]. This makes LVEF a crucial metric for assessing the function of the heart [3]. Among various heart imaging techniques, echocardiography is preferred for its quick imaging capabilities, lack of ionizing radiation, and ability to offer an immediate view of the heart in motion. In echocardiographic assessments, precisely measuring the dimensions and function of the heart is essential [4]. To compute LVEF and other vital parameters, the inner heart lining is manually outlined, a process that is labour-intensive, slow, and prone to variability. Thus, there’s an evident demand for enhancing echocardiographic evaluations through automation, focusing on the following objectives: auto-detection of end-diastolic frames (EDFs) and end-systolic frames(ESFs) during the cardiac cycle, automatic segmentation of the left ventricle, and automated calculation of left ventricular EDV and ESV.

Conventional methods for segmenting the left ventricle have utilized various approaches, such as thresholding, detecting edges, growing regions, matching templates, and using machine learning techniques. For example, Goshtasby et al. [5] developed an intensity thresholding algorithm to extract the endocardium. Leclerc et al. [6] employed a structured random forest algorithm to precisely segment the myocardium and left ventricle by using contextual information at various scale. Belous et al. [7] proposes a fully automated segmentation technique using deep learning within a Bayesian nonparametric framework, leveraging a dynamic statistical shape model from weighted training shape subsets. Some studies have worked on segmentation-based methods for the prediction of the left ventricular volume. One such study was presented by Cousty et al. [8] by segmenting the left ventricular myocardium using a watershed algorithm and evaluated the relationship between left ventricular ejection fraction (LVEF) and myocardial mass. Whereas some studies focus on directly predicting the LV volume without segmentation. Afshin et al. [9] proposed the prediction of LV volume directly by including the statistical feature analysis and support vector machine to forecast ventricular volume. However, it suffered from a lack of accuracy and stability. Wang et al. [10] proposed a method using random regression forests that directly predicted ventricular volume based on image statistics. However, these traditional techniques rely on manually designed features and adjusted parameters.

Recently, deep learning has witnessed rapid growth and has been extensively applied across a range of medical imaging areas, particularly in echocardiography [11,12]. A network known as MFP-Unet (Multi feature pyramid U Network) was introduced by Moradi et al. [13] used for the segmentation of the left ventricle and presenting a technical approach for the estimation of LVEF by detecting the long axis and ventricle area through the use of a smallest enclosing triangle. Furthermore, Liu et al. [14] unveiled a deep pyramid local attention network (PLA-Net) aimed at enhancing feature representation by effectively capturing information from adjacent contexts, both compact and sparse. Additionally, Guo et al. [15] incorporated a channel attention mechanism and introduced two segmentation networks; one focused on segmenting the left ventricle and the other targeted at segmenting the apical triangle.

EchoNet-Dynamic was brought forward by Ouyang et al. [16] in 2020, representing the most extensive dynamic echocardiography database at the time, containing 10,030 videos. They suggested a novel beat-to-beat evaluation method utilizing the DeepLab v3 architecture, which showed promising outcomes in crucial tasks like segmenting the left ventricle and estimating LVEF [16]. Moreover, Reynaud et al. [17] introduced a residual auto-encoder network, leveraging the Transformer architecture to directly predict LVEF, achieving a mean absolute error (MAE) of 5.95%. Nevertheless, the approach of directly predicting LVEF might not fully integrate into clinical workflows [18], where LVEF estimation is conventionally performed following the manual outlining of the left ventricular contour in clinical settings. Deep learning approaches have shown promise in analysing echocardiograms but face two major obstacles: poor quality of clinical echocardiograms and a lack of large-scale studies with dynamic data. Issues such as poor contrast, motion blur, incomplete edges, respiration and tangential views complicate accurate left ventricle segmentation. Additionally, the shortage of high-quality annotated datasets limits the development of effective algorithms. Synthetic data has emerged as a valuable resource for enhancing deep learning models and data augmentation, allowing for the creation of diverse datasets. Combining real and synthetic images to train medical algorithms is a successful strategy to address the lack of medical images using Generative Adversarial Networks (GANs). This research introduces an innovative method for enhancing echocardiograms using GANs for image augmentation and an automated analysis process. Rule-based systems [19] rely on predefined heuristics and expert knowledge, making them interpretable but less adaptable to complex image variations. In contrast, GANs generate realistic synthetic images, improving segmentation accuracy. A notable innovation is the MultiResUNet Architecture, which refines segmentation by modifying the traditional UNet framework. MultiResUNet uses an Inception-style block instead of dual convolutional layers, allowing detailed extraction of spatial characteristics. It processes encoder features with convolutional layers before integrating them with decoder features, unlike the conventional direct concatenation method.

The paper highlights the improved accuracy in left ventricular segmentation through GAN-based image augmentation and the MultiResUNet framework, validated using the EchoNet-Dynamic dataset and a synthetic dataset created with GAN technology. Key contributions include an automated deep learning technique for echocardiogram analysis, a workflow utilizing GANs to produce synthetic echocardiograms with accurate labels, and the MultiResUNet framework that enhances segmentation accuracy and efficiency.

## 2. Related Work

A key advancement in the area of LV segmentation was achieved with the successful integration utilizing deep learning methodologies, which resulted in the extraction of features at multiple scales for a variety of tasks. Table 1 showcases the effectiveness of earlier deep learning models that have been applied to the task of segmenting the left ventricle in echocardiography images.

Among the notable techniques listed, CETUS stands out for employing an active contour method with mathematical fitting, achieving a remarkable Dice coefficient of 0.937. Meanwhile, UCSF (University of California San Francisco) utilized a convolutional neural network (CNN) within the conventional U-Net framework, demonstrating a commendable Intersection over Union (IoU) score of 0.891. Notably, CETUS reappears with a different approach, combining an active snake technique enhanced by a CNN encoder, yielding modified Dice coefficients for end-diastole (ED) and end-systole (ES) phases. Additionally, a dataset comprising 1500 videos employed a CNN based on U-Net architecture, supplemented by Kalman filtering, reporting Dice coefficients of 0.870 and 0.860. EchoNet-Dynamic and CAMUS approaches also showcased impressive performance, with Dice coefficients ranging from 0.903 to 0.951, utilizing various CNN architectures and innovative design elements such as residual blocks and Transformer encoder bridges. Furthermore, EchoNet-Dynamic variants introduced novel strategies such as incorporating auto-encoders and attention mechanisms, resulting in Dice coefficients exceeding 0.91.

## 3. Data

The EchoNet-Dynamic dataset, accessed on 10 February 2023, via https://echonet.github.io/dynamic/index.html, contains 10,030 2D echocardiography videos from individual patients, captured from the apical four-chamber (A4C) perspective. The dataset is divided into 7465 training videos, 1288 validation videos, and 1277 testing videos.

Each video, processed into a 112 × 112-pixel clip, includes frames capturing the heart’s end-systole (ES) and end-diastolic (ED) phases. This dataset provides detailed 2D coordinate pairs mapping the left ventricle (LV) volume and shape, used as inputs for model training and evaluation. The dataset’s 10,030 videos at 112 × 112 resolution are deconstructed into individual frames with predefined masks, crucial for training and evaluating the newly introduced model. This strategy of transforming videos into frames, guided by existing masks, is influenced by the methodology outlined by Ouyang et al. [16].

## 4. Methodology

**Figure 1:**
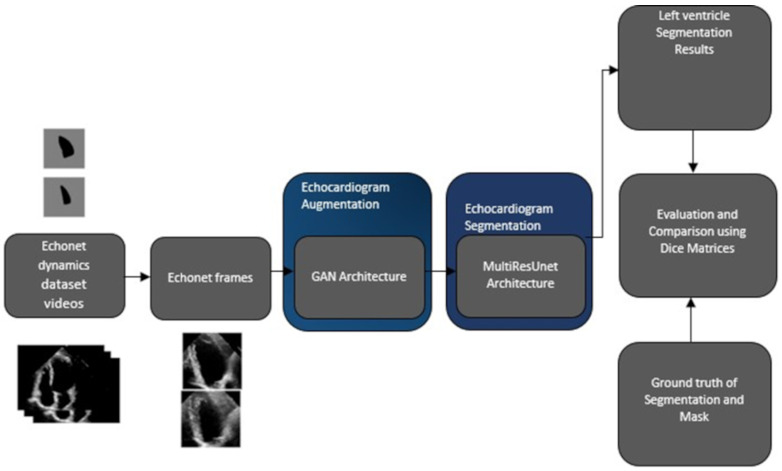
Procedural framework of the Methodology.

We conducted a comprehensive methodology to achieve accurate left ventricle segmentation from echocardiogram data. Figure 1 illustrates the steps employed. We began by collecting and curating a dataset of echocardiogram videos from EchoNet-Dynamic dataset, ensuring diverse representations of cardiac dynamics and pathologies. We extracted individual frames from the Echonet dynamics dataset videos, forming a sizable collection of images for subsequent analysis. Expert annotations label these frames in each echocardiogram, subsequently serving as the input frames for the videos.

We applied a Generative Adversarial Network (GAN) architecture to augment the dataset and improve its diversity. This allowed us to generate synthetic images closely resembling real echocardiogram frames. These synthetic images were associated with appropriate labels. We applied a deep learning model called MultiResUNet for performing left ventricle segmentation. We trained this architecture on both the real and synthetic image datasets. The segmentation results included pixel-wise delineation of the LV boundaries in the echocardiogram images. To evaluate our segmentation accuracy, we compared our findings with the annotations from the ground truth. The Dice similarity coefficients and Intersection over Union were calculated to assess the degree of overlap between the segmented regions and the manually annotated ground truth masks. Simultaneously, accuracy metrics are computed by contrasting the outcomes from the expert annotations and the algorithmic outputs. This comparison serves to quantify the performance of the two models.

### 4.1. Data Augmentation with GAN

Generative Adversarial Networks (GANs) [27] involve two components: a generator and a discriminator. The generator aims to create samples that mimic the training data, while the discriminator evaluates these samples to distinguish between real and fake ones. The generator’s goal is to produce data indistinguishable from authentic data, matching the training data distribution.

**Figure 2:**
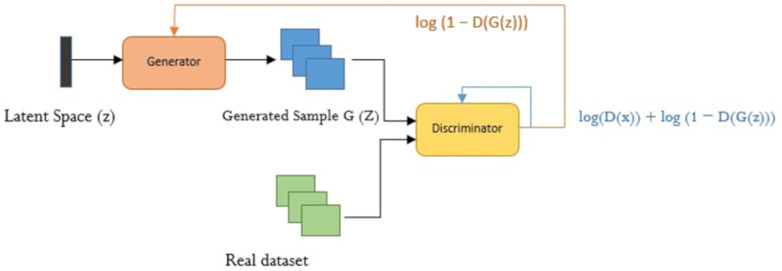
GAN Architecture.

Figure 2 illustrates the standard GAN setup. The generator selects a random point *z* from the latent space and generates G(z). The discriminator evaluates G(z) alongside a real sample, rating each as authentic (1) or fake (0). These evaluations assess both components’ performance. The generator’s goal is to minimize log(1−D(G(z))), making generated images indistinguishable from real ones (D(G(z))→1). Conversely, the discriminator aims to optimize log(D(x))+log(1−D(G(z))), enhancing its ability to differentiate real samples (D(x)) from generated ones (D(G(z))). This concurrent training sharpens the discriminator’s skills.

### 4.2. Segmentation Architecture

**Figure 3:**
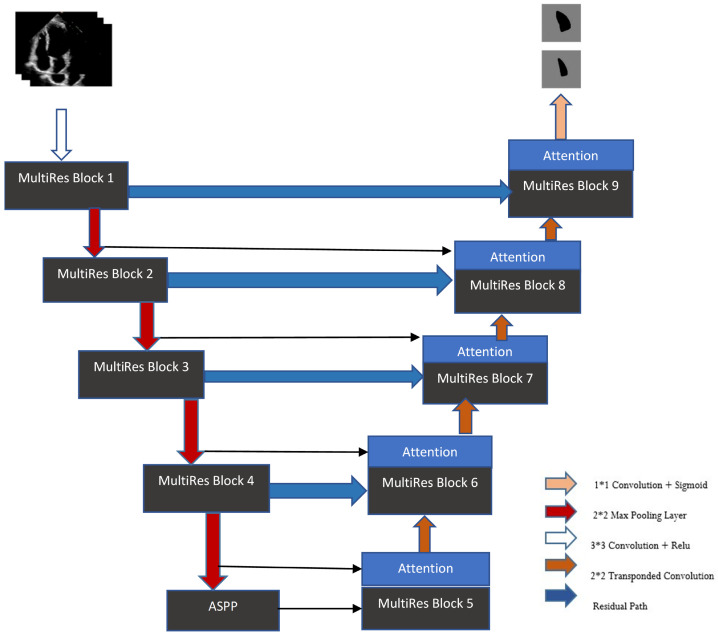
The architecture of the MultiResUNet Model.

The MultiResUNet is an advanced U-Net architecture with an encoder and a decoder. The encoder encodes input data, while the decoder reconstructs images by merging feature maps from the encoder. MultiResUNet features the MultiRes Block and the Residual Path. The MultiResUNet framework is distinguished by its dual-component structure, comprising the MultiRes block and the residual path [28]. The MultiRes Block uses parallel convolutions (Figure 3) to enhance spatial feature extraction at varied scales, balancing computational demand and precision. The proposed model enhances MultiResUNet by adding ASPP (Atrous Spatial Pyramid Pooling) blocks and attention mechanisms in each MultiRes Block within the decoder. Transposed convolution plays a critical role in the decoder stage, where it is used to upsample feature maps back to the original resolution. The proposed architecture consists of the following:

#### 4.2.1. MultiRes Block

**Figure 4:**
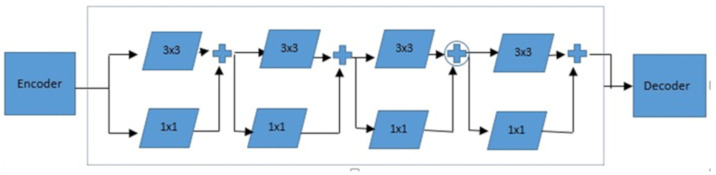
ResPath Block Structure.

The introduction of a multires block, aimed at addressing the scale variation problems in object segmentation, borrows its concept from the inception network. This block utilizes three different sizes of convolutional kernels: 3 × 3, 5 × 5, and 7 × 7. We incorporated the technique developed by Ibtehaz and Rahman [28], where the 5 × 5 and 7 × 7 kernels were substituted with 3 × 3 kernels with varying filters. Three distinct convolutional blocks are merged to capture spatial features at various scales. The number of filters in a multires block is controlled by parameter W, which multiplies the filters at each stage. Initial filters for each successive layer are set at 32, 64, 128, and 256. Filter values for multires kernels are determined by coefficients: W/6 for the first 3 × 3 kernel, W/3 for the second, and W/2 for the third. For instance, in multires block 1 with a filter value of 32, the filter values are 5.33, 10.67, and 16. Additionally, 1 × 1 convolutional layers in multires blocks enhance spatial understanding, as shown in Figure 4.

#### 4.2.2. ResPath

Ibtehaz and Rahman [28] proposed a strategy addressing the potential semantic disconnections caused by traditional skip connections linking encoders and decoders. They suggest replacing these conventional skip connections with a residual pathway, which combines feature maps from both the encoder and the decoder. In this approach, the encoder’s output passes through a convolutional layer with residual connections. In the convolutional layer, a filter with dimensions of 3 × 3 is employed. However, within the residual connections, a filter with dimensions of 1 × 1 is utilized, as depicted in Figure 4. Residual connections maintain gradient flow and stabilize training, whereas 1 × 1 convolutions aid in dimensionality reduction and feature transformation.

#### 4.2.3. SELU Activation Function

The original MultiResUNet architecture uses the Rectified Linear Unit (ReLU) as its activation function. Although ReLU enables fast computation for positive inputs, it outputs zero for non-positive inputs, potentially hindering neuron learning [29]. To mitigate this, we adopt the Scaled Exponential Linear Unit (SELU) as the activation function, defined by the following equation:

(1) $$\mathrm{SELU}\left(x\right)=\left\{\begin{array}{cc} x & \mathrm{if}\;x>0,\\ \alpha e^{x}-\alpha & \mathrm{if}\;x\leq 0, \end{array}\right.\;\mathrm{where}\;\alpha \approx 1.6732632423$$

#### 4.2.4. ASPP

The incorporation of ASPP facilitates the extraction of features across multiple scales. Atrous convolution enables precise control over the field of view, essential to capture information at multiple scales. Consistent with prior work [30], the ASPP module is applied in this study as an integral component of the bridge uniting the encoder and decoder. The governing equation for atrous convolution is:

(2) $$y\left[i\right]=\sum_{k}x\left[i+r\cdot k\right]w\left[k\right]$$

where y[i] is the output feature map, x[i] is the input, w[k] is the convolution kernel, *r* is the atrous rate, and *k* is the kernel index. ASPP consists of a 1×1 convolution for fine spatial details, three 3×3 atrous convolutions with increasing dilation rates (e.g., 6, 12, 18), and a global average pooling branch for capturing global context. These features are concatenated and processed via another 1×1 convolution, followed by batch normalization and activation. ASPP was chosen over other methods like fully connected CRFs, PSPNet, or DeepLab variants without ASPP due to its effective multi-scale context capture, improved performance in semantic segmentation, and computational efficiency. Unlike traditional convolutions, ASPP enhances dense prediction tasks by addressing fixed receptive field limitations while remaining more efficient than CRFs and other iterative post-processing techniques. In this context, it addresses the size disparity between systole and diastole segmentation objects by offering multi-scale information.

#### 4.2.5. Attention

In the field of segmentation tasks utilizing U-net, Jha et al. [30] incorporated attention blocks into the decoder that connect to the encoder. Such an architecture allowed the encoder to encapsulate all relevant data from the polyp image into a fixed-dimension vector. A significant benefit of employing an attention mechanism is its flexibility with different input sizes and ability to boost model performance by concentrating on essential parts of the feature map. Consistent with this methodology, our research adds an attention block to the decoder, connected to the encoder, thereby enhancing the model’s ability to identify the area of the left ventricle accurately.

### 4.3. Evaluation Matrices

Metrics for evaluating echocardiogram segmentation are crucial for assessing the accuracy of automated image analysis algorithms. The Dice coefficient and Intersection over Union (IoU) are commonly used metrics in this field. The Dice coefficient evaluates spatial overlap between the algorithm’s output and the ground truth, ranging from 0 to 1, where higher scores indicate better alignment. IoU measures the overlap proportion relative to the combined area of predicted and actual segmentations, ranging from 0 to 1. These metrics are vital in echocardiogram analysis, helping to develop precise and clinically relevant segmentation algorithms for cardiac diagnostics and research. Both metrics provide quantitative insights into segmentation performance, aiding in refining automated systems for tasks like left ventricle and atrium segmentation and myocardium delineation.

## 5. Experiment and Results

### 5.1. GAN Architecture

**Figure 5:**
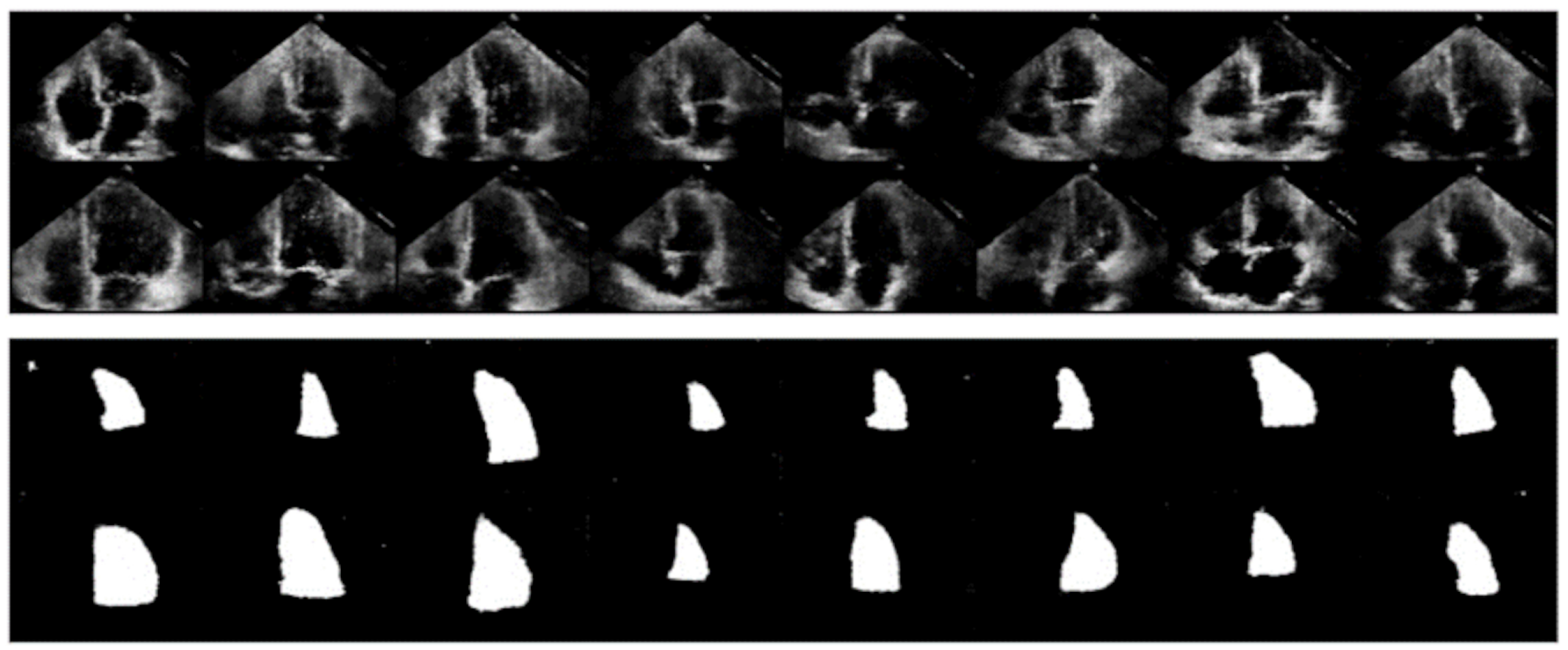
Synthetic images with corresponding masks Generated through GAN.

The GAN model’s generator uses convolutional transpose layers to up sample input noise vectors and noisy labels, generating synthetic echocardiogram images and masks. The network employs LeakyReLU activation functions, dropout layers for improved learning, and injected noise to encourage diversity in generated samples. The discriminator, designed to assess the realism of synthesized images and masks, consists of convolutional layers forming a deep hierarchical feature extractor. It evaluates image realism and assigns anatomical labels guided by binary cross- entropy and cross-entropy loss functions. Key hyperparameters include a noise vector size of 100 for sample diversity and learning rates of 0.00002 for both the generator and discriminator, with Adam optimization. Dropout layers with a 0.5 rate introduce regularization to mitigate overfitting. The loss function for training includes cross-entropy loss for anatomical label prediction and binary cross-entropy loss for image realism in the discriminator. The generator’s loss function also includes binary cross-entropy loss to ensure realistic sample generation and cross-entropy loss to align generated labels with noisy input labels. Training spans 200 epochs, using the Adam optimizer for efficient convergence. The generated echocardiogram images and corresponding masks using the described GAN architecture exhibited notable quality (Figure 5). The training began with a Discriminator loss of 175.3207 and a Generator loss of 86.8098 during the first epoch. However, as training progressed, an improvement was seen in the model performance. By epoch 200, the Discriminator loss had reduced to 132.4445, while the Generator loss had decreased to 61.8812. This loss reduction suggests that the GAN had learned to generate more convincing and anatomically accurate echocardiogram images and masks.

Furthermore, masks predicted by the GAN for generated images indicated that the GAN successfully incorporated anatomical information into the generated images. The training generated a dataset of 20,000 echocardiogram images and their corresponding masks. These synthetic echocardiograms and masks were subjected to rigorous inspection, and the findings indicated that they exhibited significantly improved quality compared to the original dataset, capturing the intricate anatomical details and textures characteristic of echocardiogram images.

### 5.2. Segmentation Architecture (MultiResUnet)

#### 5.2.1. Dataset

Our dataset comprises 10,030 echocardiogram images with corresponding masks, meticulously sourced from the Echonet Dynamics database, ensuring the availability of high-quality ground truth annotations. Additionally, we introduced a novel aspect to our research by generating an additional 10,000 synthetic echo images, complete with corresponding masks, using a Generative Adversarial Network (GAN) architecture. After GAN-based synthetic augmentation, the dataset size doubled, leading to an increased distribution of 14,930 training, 2576 validation, and 2524 test samples. Table 2 shows the sample dataset size before and after GAN.

#### 5.2.2. Training Process

The training process for the echocardiogram left ventricle segmentation model involved a carefully selected set of hyperparameters and strategies. The Adam optimizer, chosen for its flexible learning rate capabilities, was used with a learning rate $1\times 10^{-3}$ and batch size 32. We incorporated dropout layers with a rate of 0.5 in both the GAN and MultiResUNet architectures for regularization. To enhance training data diversity, we applied traditional augmentation techniques such as rotation, flipping, and scaling. Additionally, we used early stopping based on validation loss and performed cross-validation to improve generalization. The training set was shuffled before each epoch to expose the model to diverse samples and avoid biases. The loss function combined Binary Cross-Entropy Logit Loss (BCE Logit Loss) and Dice Loss. BCE Logit Loss guided accurate pixel-wise classification, while Dice Loss encouraged precise boundary delineation. Training spanned 50 epochs, chosen to balance convergence and prevent overfitting, with model checkpoints saved every 5 epochs. Training on a CUDA-enabled GPU leveraged hardware acceleration for faster training times and efficient resource utilization.

#### 5.2.3. Evaluation Metrics

We adopted the Dice coefficient index and intersection over union (IoU) to measure our model’s performance. These metrics were computed by comparing the predicted LV region (*S*) to the ground truth LV segmentation results provided by human experts from the EchoNet-Dynamic dataset (SE). The equations for IoU and Dice coefficients are:

(3) $$\begin{array}{c} \mathrm{IoU}=\frac{\mathrm{Area}\;\mathrm{of}\;(S\cup SE)}{\mathrm{Area}\;\mathrm{of}\;(S\cap SE)}\;\\ \mathrm{Dice}\;\mathrm{Coefficient}=\frac{2\cdot |S\cap SE|}{P(S)+P(SE)} \end{array}$$

where, P(S) and P(SE) represent the probabilities that a pixel belongs to the predicted segmentation and the expert-annotated ground truth, respectively. A higher Dice Coefficient value indicates better alignment with the ground truth.

### 5.3. Implementation Results of Segmentation Architecture

The results of our study employing the MultiResUNet architecture for left ventricular segmentation in echocardiograms have been highly promising and demonstrate the model’s substantial potential for clinical applications:

#### 5.3.1. Training Phase

**Figure 6:**
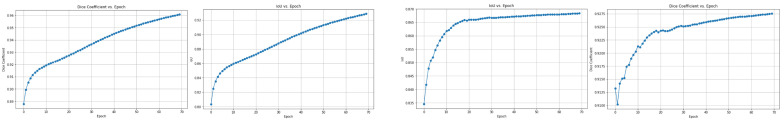
Dice Coefficient vs. Epoch and IoU vs. Epoch Graph during training (**left two plots**) and validation (**right two plots**), respectively.

The dice coefficient, accuracy, and IoU steadily increased during training, showing consistent improvement. Precision, recall, and F1 scores remained high, indicating a balanced trade-off between true and false positives and negatives. To assess the functionality of the models, we closely tracked the training process’s loss and the IoU and Dice scores during each validation step. During the training process, there was a notably more substantial improvement in the loss. The model demonstrates remarkable progress across 70 epochs, with the dice coefficient steadily increasing to an impressive 0.9607. This signifies the model’s ability to capture the intricate details of the left ventricle, leading to higher accuracy of 99.27, precision of 88.64, recall value 89.57, F1 score 89.10, and IoU 92.85. Figure 6 (left images) provide the training graphs of Dice coefficient and IoU with respect to the epochs.

#### 5.3.2. Validation Phase

Moving on to the validation results, the MultiResUNet architecture maintains its high performance, with a dice coefficient of 0.9289 and an accuracy of 0.9862 in the final epoch. This underscores the model’s generalization capability, performing well on unseen data. The accuracy 98.62, precision 87.90, recall 94.28, F1 score 90.98, and IoU 86.83 also remain consistently high, suggesting that the model’s segmentation predictions are well-balanced and robust. Validation results corroborated the model’s robustness, showcasing consistent and positive trends in evaluation metrics. Figure 6 (right images) provide the validation graphs of Dice coefficient and IoU with respect to the epochs.

#### 5.3.3. Testing Phase

**Figure 7:**
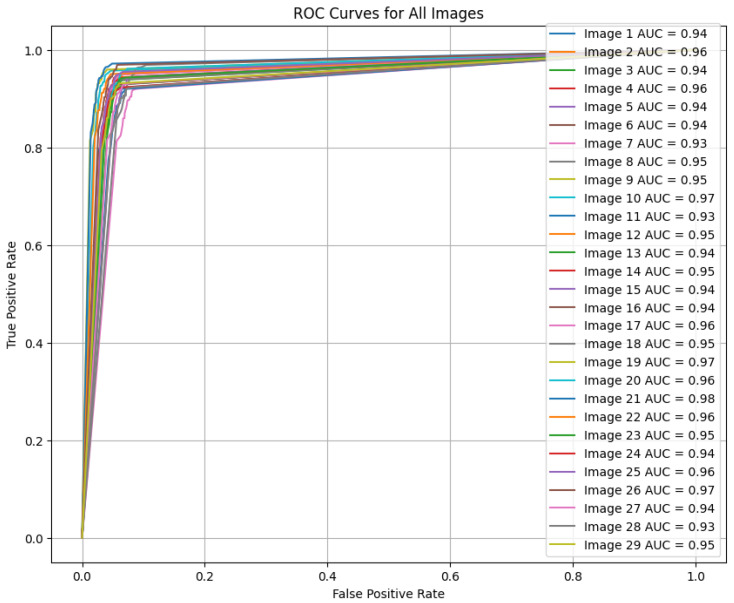
ROC curve for all test images.

The most crucial evaluation comes from the testing phase, where the model achieves good results. The dice coefficient of 0.9568 indicates a high intersection between the predicted and actual truth left ventricular regions. The accuracy 99.76, precision 98.98, recall 98.60, F1 score 98.79, and IoU 91.62 are also high, showcasing the model’s ability to accurately delineate the left ventricle in echocardiograms. These results are especially critical in a medical context, where precise segmentation can aid in diagnosing heart conditions and guiding treatment decisions. Figure 7 shows the ROC curve for all the test images, with the AUC value being close to 1 for most of them.

#### 5.3.4. Comparison of Other Methods

The standard deep learning models were trained on the EchoNet-Dynamics dataset and compared with the proposed approach. Table 3 presents a comparative analysis of various deep learning architectures applied to left ventricular segmentation in echocardiograms. The evaluation includes key metrics such as the Dice coefficient, Jaccard index, precision, accuracy, F1 score, and area error ratio. These metrics assess the effectiveness and efficiency of each model in segmenting the left ventricle from echocardiographic images. The results highlight the proposed approach’s superior performance in achieving higher accuracy and precision, thereby demonstrating its potential for improving diagnostic accuracy in echocardiographic analysis.

#### 5.3.5. 2D Projection with LOF Anomaly Detection

**Figure 8:**
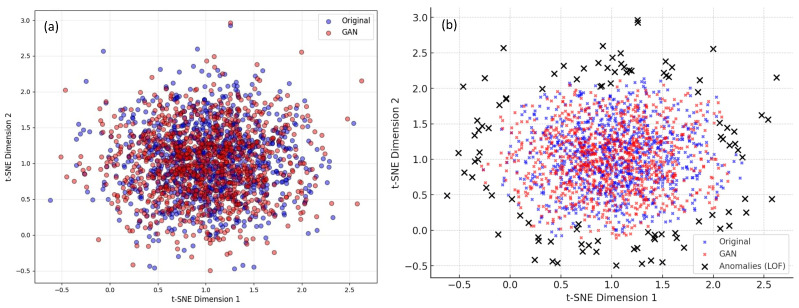
(**a**) 2D projection plot of high-dimensional image features before and after GAN-based approach (**b**) Local Outlier Factor (LOF) anomaly detection. The blue points correspond to the original dataset, while the red points represent the synthetic images generated by the GAN. The black markers represent outliers detected using LOF.

The t-SNE visualization shown in Figure 8a demonstrates the 2D projection of high-dimensional image features before and after augmentation using a GAN-based approach. In this plot, blue points correspond to the original dataset, while red points represent GAN-generated synthetic images. The strong overlap between these points indicates that the GAN effectively captures the distribution of real data without introducing significant shifts. Additionally, the dense central region suggests that the generated samples align closely with the original dataset, reinforcing the model’s ability to learn essential patterns. However, a few outliers, represented by scattered red points at the periphery, hint at minor variations in GAN-generated images, potentially due to mode collapse. To further analyze this, we applied Local Outlier Factor (LOF) anomaly detection to highlight deviations in the dataset. In Figure 8b black ‘x’ markers in visualization represent outliers detected by LOF, primarily concentrated at the edges. These anomalies appear in both real and GAN-generated samples, suggesting that the dataset naturally contains outliers rather than the GAN exclusively producing them. To refine the synthetic dataset, we applied LOF-based filtering, removing detected anomalies before training a MultiResUNet + ASPP + Attention model. This filtering process resulted in a slight improvement in performance, as evidenced by increased Dice Coefficient, IoU, and F1-Score shown in the Table 4. The results confirm that LOF-based anomaly detection enhances GAN-augmented data quality, improving model generalization.

## 6. Discussion

**Figure 9:**
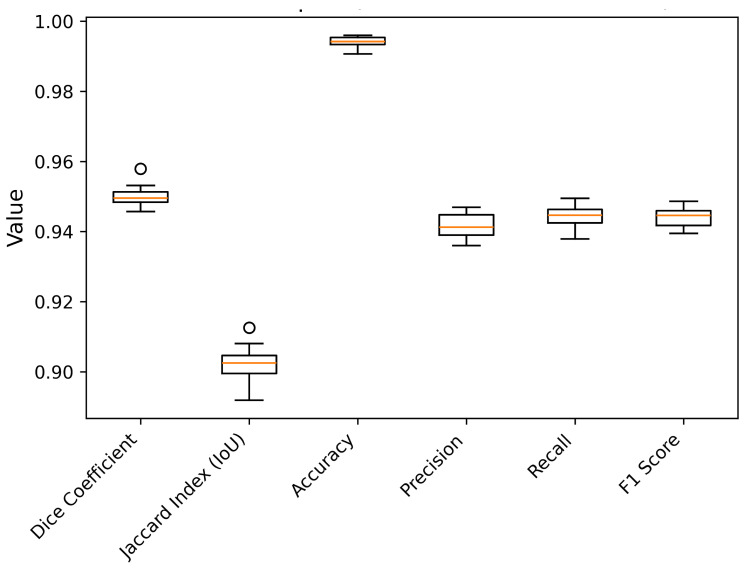
Boxplots of 10 fold cross validation results of proposed approach.

The high training Dice score (96.07%) shows effective learning, while the slightly lower validation score (92.89%) indicates good generalization with room for improvement. The high-test score (95.68%) confirms excellent generalization to new data, highlighting its clinical potential. The analysis of the training, validation, and test results reveals a notable trend in the performance of the proposed model for left ventricular segmentation in echocardiograms. Notably, the test results exhibit significantly superior performance in terms of both the dice coefficient and IoU compared to both the training and validation phases. This divergence indicates that the model generalizes extraordinarily well to previously unseen data in addition to learning from the training set of data. We also evaluated 10-fold cross validation of proposed model and Figure 9 shows the box plots of each metrics. These results indicate that the model performs consistently across different training subsets, as evidenced by low standard deviations across all metrics. The high correlation with our testing phase metrics (Dice: 95.68%, IoU: 91.62%) further confirms robust generalization with minimal bias. While slight variations exist due to dataset composition, the low variance across folds ensures that the model is not overly dependent on any specific training subset, reinforcing its real-world reliability.

Table 5 comprehensively compares various deep learning architectures applied to the same dataset, EchoNet Dynamics, highlighting their performance regarding the Intersection over Union (IoU) and Dice Coefficient. Notably, in this competitive landscape, the MultiResUNet model, proposed in this research, emerges as a standout performer. With good IoU and Dice Similarity Coefficient scores of 91.62% and 95.68%, respectively, it surpasses the results obtained by previous state-of-the-art models, including DeepLabV3, TransBridge, Trans U-net, Swin Transformer, Segformer Network, and MAEF-Net.

Among the notable models, Minqi Liao et al. [11] have exhibited commendable results utilizing innovative approaches like Swin Transformer, K-Net, and Segformer Network. Their contributions, with Dice coefficients ranging from 92.79% to 92.92%, signify the effectiveness of these sophisticated architectures in accurately segmenting the left ventricular region. Additionally, Yan Zeng et al. [31] presented the MAEF-Net, achieving an impressive Dice coefficient of 93.10%, further demonstrating the continual advancements in cardiac image segmentation. However, it is crucial to note that while these models achieved remarkable results, they did not incorporate synthetic data augmentation.

**Figure 10:**
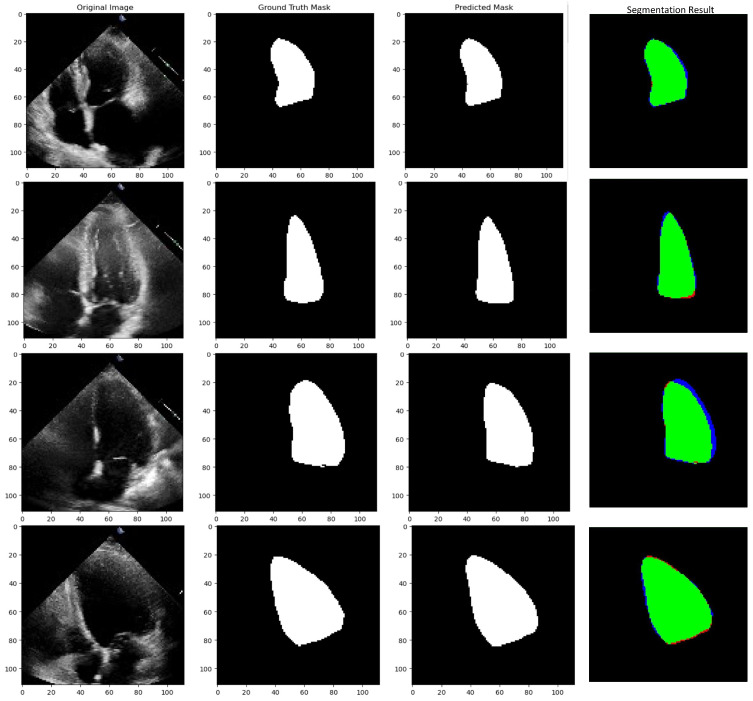
MultiResUNet result vs. Expert Annotated LV Segmentation. Green represents true-positive pixels, red indicates false-positive pixels, and blue highlights false-negative pixels.

This study not only illustrates the potential of innovative architectures but also accentuates the transformative impact of data augmentation techniques, particularly in cardiac image analysis, paving the way for more accurate and reliable clinical applications. Figure 10 below is the predicted left ventricular segmentation vs. expert annotation of LV segmentation.

Our work not only introduces the MultiResUNet architecture but also harnesses the power of GANs to generate synthetic data, effectively doubling the size of the dataset. This approach has led to a significant boost in segmentation accuracy, indicating the substantial impact of data augmentation in overcoming dataset size and diversity limitations.

Additionally, Integrating GAN-generated synthetic data significantly enhanced the performance of our segmentation model. We conducted a comprehensive evaluation to assess the quality and utility of the GAN’s synthetic echocardiogram images and masks generated. This evaluation involved both qualitative and quantitative analyses. Qualitatively, the synthetic images were subjected to rigorous inspections by clinical experts, who confirmed that the images exhibited high anatomical fidelity and closely resembled real echocardiograms. Quantitatively, the segmentation performance of the MultiResUNet model trained with both real and synthetic data was compared to a model trained exclusively with real data. The results showed a substantial improvement in segmentation accuracy, with the model achieving a Dice similarity coefficient of 95.68% and an IoU of 91.62%. Further, the integration of LOF with the GAN approach led to a slight improvement in performance, as evidenced by Table 4. This generation of synthetic data would be helpful in addressing the limitations of small and diverse training datasets.

Despite the excellent outcomes attained by the MultiResUNet model and the integration of GAN-based synthetic data augmentation, several limitations must be acknowledged. Firstly, although comprehensive, the reliance on the EchoNet-Dynamic dataset may not fully capture the diversity of real-world clinical scenarios. This data utilises LV segmentation in a single frame and therefore would not be a good predictor of EF in patients with arrythmias, who would require averaging of EF over 5 beats. This limitation could impact the model’s generalizability when applied to different populations or imaging conditions.

## 7. Conclusions and Future Work

This work advances left ventricular segmentation in cardiac images with the MultiResUNet architecture. Evaluated on the EchoNet Dynamics dataset, the model achieved a Dice coefficient of 95.68% and an IoU of 91.62%, showing strong generalization to unseen data. GAN-generated synthetic data, doubling the dataset size, significantly enhanced model performance. This underscores the value of synthetic data in medical imaging. Compared to models like DeepLabV3, ResNet, and Trans U-net, MultiResUNet achieved superior accuracy, setting a new standard in left ventricular segmentation. Overall, this research significantly advances cardiac image analysis, offering accurate segmentation and promising improvements in clinical decision-making and patient care.

Future work may focus on refining the MultiResUNet architecture, improving segmentation accuracy, and evaluating its adaptability to other cardiac imaging modalities such as MRI and 3D echocardiography. Integrating multi-modal information, such as ECGs and patient history, with cardiac image data can enhance the model’s understanding of cardiac anatomy and function, leading to more accurate clinical assessments.

## Figures and Tables

**Table 1:** Deep learning-based studies.

Bibliography	Dataset	Methods	Achieved Results
[3]	CETUS (MICCAI Challenge Dataset)	Utilized an active contour method with mathematical fitting	Achieved a Dice coefficient of 0.937
[20]	UCSF	Employed a Convolutional Neural Network within the conventional U-Net framework comprising 23 layers	Attained an IoU score of 0.891
[21]	CETUS (MICCAI Challenge Dataset)	Implemented an active snake technique enhanced by a Convolutional Neural Network encoder acting as a locator	Demonstrated modified Dice coefficients of 0.112 (ED) and 0.160 (ES)
[22]	1500 videos	Utilized a CNN model with U-Net architecture and supplementary training involving Kalman filtering	Reported Dice coefficients of 0.870 (CNN) and 0.860 (KF)
[23]	CETUS (MICCAI Challenge Dataset)	Employed a Convolutional Neural Network incorporating autoencoder architecture to align with the structure of the LV	Achieved Dice coefficients of 0.912 (ED) and 0.873 (ES)
[16]	EchoNet-Dynamic	Developed a Convolutional Neural Network using the Deeplab V3 architecture and atrous convolutions	Attained Dice coefficients of 0.927 (ED) and 0.903 (ES)
[24]	CAMUS DATASET	Created a Convolutional Neural Network with a combination of residual blocks and U-Net-based encoder-decoder architecture	Achieved a Dice coefficient of 0.951
[1]	EchoNet-Dynamic	Convolutional Neural Network with Transformer architecture connected with encoder and decoder	Demonstrated a Dice coefficient of 0.916
[25]	EchoNet-Dynamic (screened)	U-Net architecture with Transformer	Achieved a Dice coefficient of 0.925
[26]	EchoNet-Dynamic (screened)	EASPP module and channel-spatial dual attention mechanism with Convolutional Neural Network	Dice: 0.931 (LV)

**Table 2:** Dataset before and after GAN.

Dataset	Training	Validation	Testing
Original	7465	1288	1277
After GAN	14,930	2576	2524

**Table 3:** A comparative analysis of various deep learning architectures applied to left ventricular segmentation in echocardiograms.

Method	Dice Coefficient	Jaccard Index (IoU)	Precision	Accuracy	F1-Score	Area Error Ratio	Other Notes
UNet	0.89	0.81	0.88	0.91	0.88	0.15	Strong baseline, sensitive to noise
UNet++	0.91	0.84	0.90	0.93	0.91	0.12	Improved multi-scale segmentation
Attention-UNet	0.92	0.85	0.91	0.94	0.92	0.11	Better edge refinement
ResUNet	0.90	0.83	0.89	0.92	0.90	0.13	Efficient with residual connections
R50-AttnUNet	0.93	0.87	0.92	0.95	0.93	0.10	Uses EMA for precision
DeepLabv3+	0.94	0.88	0.93	0.96	0.94	0.09	Excellent for large datasets
YOLO-based	0.92	0.85	0.91	0.94	0.92	0.11	Optimized for speed
MultiResUNet	0.91	0.86	0.89	0.98	0.90	0.02	Ablation 1
MultiResUNet + ASPP + Attention (Without GAN )	0.92	0.87	0.91	0.98	0.91	0.02	Ablation 2
MultiResUNet + ASPP + Attention + GAN (Proposed approach)	0.96	0.92	0.99	0.99	0.98	0.02	Optimized for handling noises and variability in echocardiogram data

**Table 4:** Effect of LOF anomaly filtering on proposed model’s performance.

Model	Dice Coefficient	JaccardIndex (IoU)	Precision	Accuracy	F1-Score
Proposed Model (with GAN)	0.9568	0.9162	0.9898	0.9976	0.9879
Proposed Model (with GAN + LOF)	0.9582	0.9185	0.9901	0.9978	0.9883

**Table 5:** Table comparing various model for the LV segmentation using EchoNet-Dynamic. NA (Not Applicable) indicates that the study has not evaluated the IoU.

Authors	Methods	Year	Dataset	IoU	Dice
Ouyang et al. [16]	DeepLabV3 and ResNet	2020	EchoNet Dynamics	NA	91.50
Deng et al. [1]	Trans Bridge	2021	EchoNet Dynamics	NA	91.64
Chen et al. [25]	Trans U-net	2021	EchoNet Dynamics	NA	92.54
Minqi Liao et al. [11]	Swin Transformer and K-Net	2023	EchoNet Dynamics	86.78	92.92
Minqi Liao et al. [11]	Segformer Network	2023	EchoNet Dynamics	86.57	92.79
Yan Zeng et al. [31]	MAEF-Net	2023	EchoNet Dynamics	NA	93.10
	Proposed MultiResUnet	2024	EchoNet Dynamics and Synthetic Dataset	91.62	95.68

## Data Availability

The EchoNet-Dynamic dataset is publicly available at https://echonet.github.io/dynamic/index.html (accessed on 25 February 2024).
